# Migrant children in transit: health profile and social needs of unaccompanied and accompanied children visiting the MSF clinic in Belgrade, Serbia

**DOI:** 10.1186/s13031-021-00366-5

**Published:** 2021-04-29

**Authors:** Tijana Topalovic, Maria Episkopou, Erin Schillberg, Jelena Brcanski, Milica Jocic

**Affiliations:** 1 International Committee of the Red Cross, Tresnjinog cveta 1, Belgrade, 11000 Serbia; 2 Médecins Sans Frontières Operational Centre Brussels, Athens, Greece; 3 Public Health Agency of Canada, Ottawa, Canada; 4 National Institute of Public health of Serbia, Belgrade, Serbia; 5 Adventist Development and Relief Agency, Belgrade, Serbia

**Keywords:** Transit migration, Unaccompanied migrant children, Accompanied migrant children, Migration, Health profile, Mental health profile, Social care

## Abstract

**Background:**

Thousands of children migrate to Europe each year in search of safety and the promise of a better life. Many of them transited through Serbia in 2018. Children journey alone or along with their family members or caregivers. Accompanied migrant children (AMC) and particularly unaccompanied migrant children (UMC) have specific needs and experience difficulties in accessing services. Uncertainty about the journey and daily stressors affect their physical and mental health, making them one of the most vulnerable migrant sub-populations. The aim of the study is to describe the demographic, health profile of UMC and AMC and the social services they accessed to better understand the health and social needs of this vulnerable population.

**Methods:**

We conducted a retrospective, descriptive study using routinely collected program data of UMC and AMC receiving medical, mental and social care at the Médecins sans Frontières clinic, in Belgrade, Serbia from January 2018 through January 2019.

**Results:**

There were 3869 children who received medical care (1718 UMC, 2151 AMC). UMC were slightly older, mostly males (99%) from Afghanistan (82%). Skin conditions were the most prevalent among UMC (62%) and AMC (51%). Among the 66 mental health consultations (45 UMC, 21 AMC), most patients were from Afghanistan, with 98% of UMC and 67% of AMC being male. UMC as well as AMC were most likely to present with symptoms of anxiety (22 and 24%). There were 24 UMC (96% males and 88% from Afghanistan) that received social services. They had complex and differing case types. 83% of UMC required assistance with accommodation and 75% with accessing essential needs, food and non-food items. Several required administrative assistance (12.5%) and nearly a third (29%) legal assistance. 38% of beneficiaries needed medical care. Most frequently provided service was referral to a state Centre for social welfare.

**Conclusion:**

Our study shows that unaccompanied and accompanied migrant children have a lot of physical, mental health and social needs. These needs are complex and meeting them in the context of migration is difficult. Services need to better adapt by improving access, flexibility, increasing accommodation capacity and training a qualified workforce.

## Background

Thousands of people migrate to Europe each year in search of safety and the promise of a better life. In 2018, around 30,000 of these people were children [1]. During migration children find themselves “in transit”, *on the move* or staying only temporarily in one or more countries along their journey towards the final destination [2]. Children can journey unaccompanied or along with their family members or caregivers [2]. Daily challenges during migratory travel, affect their physical and mental health, making them extremely vulnerable [3]. Migrant children in transit, particularly if unaccompanied, are less likely to have access to health and social services of the hosting country [2]. In such setting children are hard-to-reach and often isolated, hence meeting their complex needs and problems is challenging [4]. In addition, it is in the context of transit migration when child protection services are the weakest [2, 4]. Child’s health is generally affected by lack of support, protection and substandard living conditions [5]. Uncertainty about continuation of the journey, problems in communication due to cultural and language differences add to the potential of unfavorable outcomes [5].

Serbia is one of the central transit countries along the so-called Balkan migration route. In December 2018, 1140 migrant children were present in Serbia [1]. Many of these children were unaccompanied or separated from their families. Although there is no official registry publicly available to establish the exact number of unaccompanied migrant children (UMC) in the country, it was estimated that around 500 were accommodated in the asylum and transit-reception facilities run by the government [5]. At the same time, government centers struggled to address the health and social needs of accompanied migrant children (AMC) and UMC and they provided limited to no access to social, education activities or tailored integration programs [4, 5].

To address the health and social needs of migrants in transit, in early 2017, Médecins sans Frontières (MSF) established an outpatient fixed clinic, in Belgrade city center. Approximately 35% of 23,901 patients who presented to the clinic in 2017 and 2018 were children, of which over 50% were unaccompanied. MSF recognizes children and UMC in particular, as a vulnerable group that requires specialized health and social care and has adapted its services to address the needs of this population.

So far, there have been several studies describing health profile of migrants, refugees and asylum seekers either upon arrival to Europe (mainly Greece and Italy) or after reaching destination countries such as Belgium, Germany and Switzerland [6–9]. UMC and AMC have been studied with regards to development and treatment of mental health symptoms, however there is a gap in comprehensive description of medical, mental health and social care in the context of transit migration [10].

The aim of the study is to describe the demographic and health profile of all children who received medical, mental or social care at the MSF clinic in Belgrade from January 2018 to January 2019, divided in two groups, UMC and AMC, to understand the health and social needs of these vulnerable populations. In addition, we described the package of care offered at the Clinic to identify gaps and opportunities for improved services provision.

## Methods

### Study design

This was a retrospective, descriptive study using routinely collected program data.

### Study setting

Serbia is a central country of transit along the so-called Western Balkan migration route. Despite the attempted closure of the corridor, via establishment of physical barriers across international borders, the Balkan route continues to be used by migrants attempting to reach the European Union. It is estimated that over 70,000 migrants entered Serbia in 2018 and 2019 [11, 12]. Although, this represents a decrease in entries compared to the peak of the migrant crisis in 2015, migrants reported longer stays in Serbia in 2018 and 2019 as border closures impeded their journey.

### MSF core activities in Serbia

MSF operates its core activities from a fixed location in Belgrade. It runs an outpatient clinic that provides primary health care services (diagnosis, treatment) and mental health support to new arrivals, undocumented migrants in transit, migrants transiting from government-run asylum and reception centers to borders, those who do not receive adequate care in centers and those who live outside of the officially established system.

In addition to this, MSF works in outreach with a medical mobile clinic and non-medical capacities (distribution of non-food items -NFIs, shelter) that are used to respond to the needs of populations congregating in informal areas, including the areas close to the borders of neighboring states. This activity is carried out by a small-scale team engaging from Belgrade when needed.

### Provision of social care by MSF

Apart from medical and mental health services, the outpatient clinic also provides social protection and case management services. In 2018, a social worker was added to the team to identify the social needs, in particular protection needs, of highly vulnerable patients, directly support or refer them to external social services actors and follow the progress of their situation. The social worker also facilitated and advocated for access to legal aid, social and health services, accommodation, while providing information about the available sources of support.

Specifically, MSF social services focused on delivering social-related activities, liaising with legal, administrative, medical and non-medical actors, in order to ease patient’s situation, maximize adherence to treatment, reach therapeutic goals and improve psychosocial condition.

**Fig. 1:**
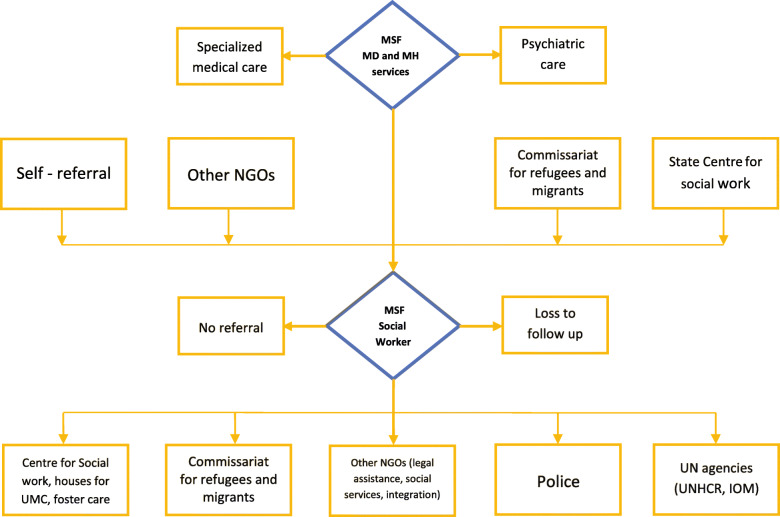
Patient flow chart of social care services provided by MSF and external actors

Figure 1 outlines the patient flow through social care services at the clinic. The most common referral pathway is via staff at the MSF clinic who identify social needs and refer patients who present for medical or mental health care. Additionally, patients can self-refer or can be referred by another NGO, Commissariat for refugees and migrants or state centers for social work. After consultation, the social worker can refer the child to one or more services such as housing, protection and legal support. The organizations presented as referral end-points in Fig. 1, provided guardianship and integration services for unaccompanied children (state Centre for Social Work, Jesuit Relief Services integration house for vulnerable refugees etc.), protection, administrative assistance including registration for new arrivals (police), protection and accommodation (Serbian Commissariat for Refugees and Migrants), legal assistance, protection, access to essential services, transportation to transit-reception and asylum centers around the country (Praxis, Info park, Belgrade Center for Human Rights, Atina etc.), resettlement and assisted voluntary return services (UNHCR and IOM). Referral pathways between different actors were often two directional as cooperation was key for complex case management.

### Study population

Study population included all children, UMC and AMC, who attended the MSF outpatient clinic in Belgrade, from January 2018 through January 2019.

“Migrant is someone who changes his or her country of usual residence, irrespective of the reason for migration or legal status” as per the International Organization for Migration [4]. Migrant children do not fall under a unique definition, however it is recognized that they may travel accompanied by parents, other adults or alone [4]. Accompaniment status of children was self-reported by the child at the time of the consultation. UMC reported traveling alone and AMC with their family members of caregivers.

### Data sources, collection and variables

Medical data was collected and directly entered into a database using the People on the move (*POM)* application. All data entered through POM application was stored in an electronic, online database. POM was an application developed by IBM, with technical inputs from MSF and donated to MSF Italy to support the response to the migration crisis in 2016. When the use of application was discontinued by the medical team, it was replaced by KoBo toolbox, an open source, free software for data collection, developed by Harvard humanitarian initiative in March 2019 [13]. The variables collected in POM and KoBo for medical patients included information on demographic characteristics of patients (sex, age, country of origin), vulnerabilities (unaccompanied minors, children under 5, disabilities, mental health condition, chronic physical illness, victims of human trafficking), main reason of consultation (disorders categorized generally by different organ systems), previous stay in the camp and access to health services along the migration route (yes/no), experience of trauma along the journey (list of traumatic events), trip duration (in months), outcome of treatment (no referral, referral accepted by patient, referral refused by patient).

The mental health consultation data and social work database were generated in excel file tables. Mental health data was collected via structured interview, conducted by a mental health professional (clinical psychologist or psycho-therapist). Due to dominant transit context and difficult follow up, no recognized assessment tools were used. Symptoms such as agitation, disturbance, uneasiness were recognized and oftentimes self-reported. Collected data was demographic (sex, age, country of origin), data on vulnerabilities (unaccompanied minor, moderate to severe mental illness, victim of sexual and gender-based violence, survivor of torture, no vulnerability), place of residence (asylum or transit-reception center, Belgrade streets/squats, private accommodation etc.) experience of trauma along the journey (list of traumatic events), trip duration (in months), major category of symptoms (symptoms of anxiety, depression, adjustment, psychosomatic symptoms, PTSD, behavioral problems etc.), sexual and gender-based violence (pre migration, peri-migration, pre and peri migration), torture (pre migration, peri-migration, pre and peri migration), referral to mental health services (MSF MD or nurse, other NGO, self-referral, friend or family, identification after group session), referral to psychiatrist (yes/no).

The social worker also conducted a structured interview and collected demographic data (sex, age, country of origin), data on vulnerability (unaccompanied minor) and case type (social protection with medical and/or mental health care), date of arrival to Serbia and leaving the country of origin, administrative status (registered, without legal administrative status), previous and current place of residence, referred by whom to social worker (MSF MD, mental health specialist, MSF cultural mediator). Also, all requests to the social worker were categorized as administrative, legal assistance, referral to a state social worker, accommodation (shelter, safe accommodation, special institution/hospital), protection from violence, UNHCR resettlement, IOM voluntary return, non-food items and food, health services, accompaniment. It was noted if the assistance was provided successfully or not and finally if further referral was needed, what was the case status (open, closed), the reason of closing the case (beneficiary left the country, lost to follow up, referral to another actor, other).

Doctors and other medical team members (nurses, cultural mediators) were trained to recognize if the patient needed mental health or social worker assistance. Access to these databases was granted only to psychologists and social worker, who collected and entered all data on a password protected laptop.

### Data analysis

Data collected from POM, mental health and social work databases during the study period was imported in MS Excel 2010 version. Variables used for research were summarized using descriptive statistics, frequencies and proportions. The Chi-square test was used to assess the significant difference between the UMC and AMC populations in the category of medical conditions (main reasons for consultation). All analyses were completed using STATA version 11 (Stata Corp. LLC, Texas, USA).

### Ethics

This research fulfilled the exemption criteria set by the Médecins Sans Frontières Ethics Review Board for a posteriori analyses of routinely collected clinical data and thus did not require MSF ERB review. It was conducted with permission from Medical Director, Operational Centre Brussels, Médecins Sans Frontières. In addition, approval was officially granted by the Ethics board of the National Institute of Public health of Serbia.

## Results

### Profile of patients treated at the medical department of Belgrade clinic, from January 2018 to January 2019

**Fig. 2:**
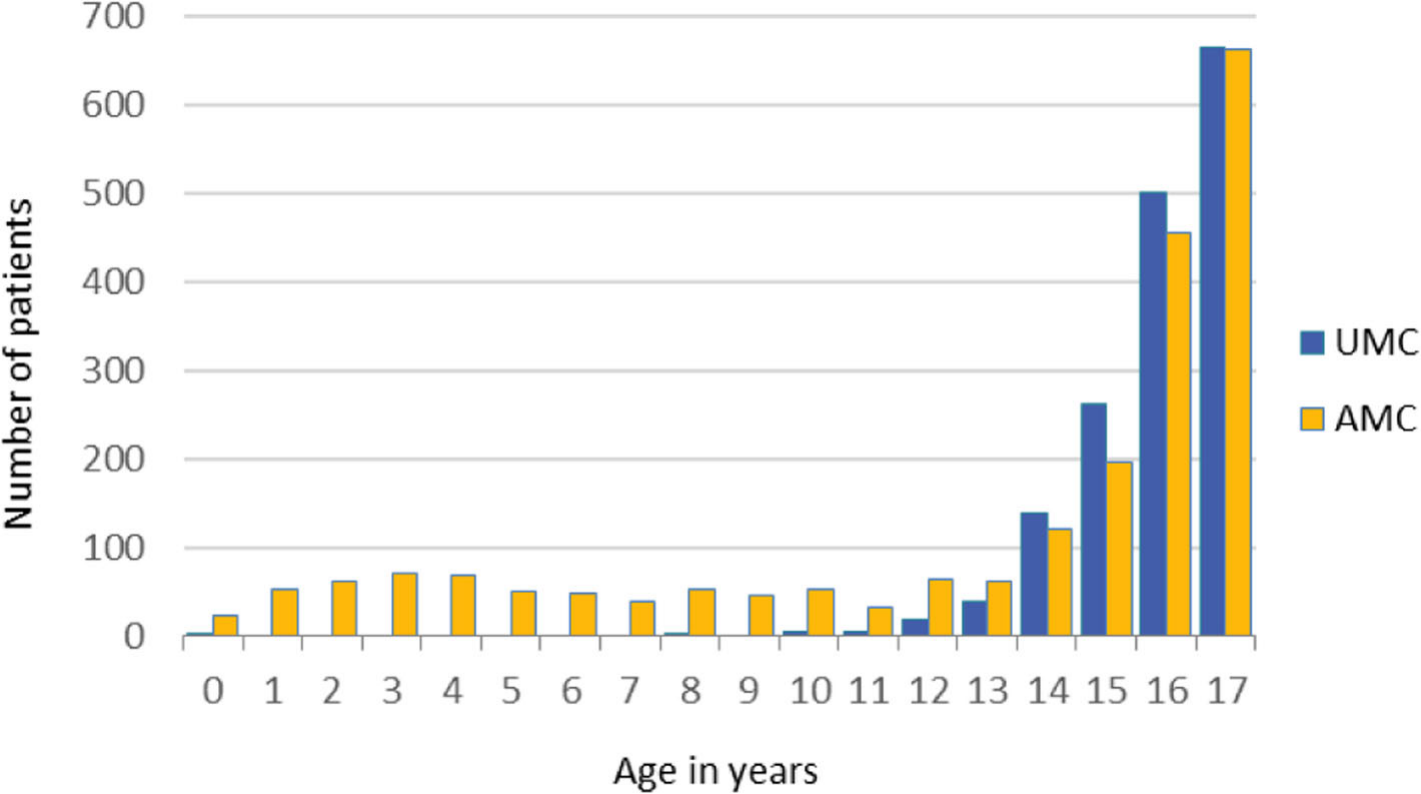
Age distribution of unaccompanied and accompanied migrant children coming for a medical consultation at Belgrade outpatient clinic, January 2018 to January 2019

A total of 1718 unaccompanied and 2151 accompanied children received medical consultations during the study period. Median age for both groups was 16 (IQR 15–17), mean 14.5. Among UMC 98% (1686/1718) were over the age of 12 compared to 69% (1493/2151) of AMC (Fig. 2). UMC were more likely to be male (Table 1). Afghanistan (82%) followed by Pakistan (12%) were the most common countries of origin of UMC, whereas for AMC it was Afghanistan (67%) followed by Iraq (13%).

**Table 1:** Demographic and health characteristics of unaccompanied and accompanied migrant children treated at the Medical department of Belgrade clinic, from January 2018 to January 2019

MEDICAL CARE	UMC	AMC
	N (%)	N (%)
**SEX**	1718 (100)	2151 (100)
Male	1704 (99)	1843 (86)
Female	14 (1)	308 (14)
**AGE** median (IQR)	16 (15–17)	16 (9–17)
**COUNTRY OF ORIGIN**		
Afghanistan	1402 (82)	1439 (67)
Pakistan	209 (12)	158 (7)
Algeria	22 (1)	22 (1)
Lybia	21 (1)	19 (1)
Iraq	20 (1)	270 (13)
Iran	12 (1)	147 (7)
Others	31 (2)	96 (4)
Missing data	1 (0.1)	0
**TRIP DURATION in months** median (IQR)	6 (0–10)	6 (1–12)
**MAIN REASONS FOR CONSULTATION**	1718 (100)	2151 (100)
Skin diseases	1059 (62)	1125 (51)
Respiratory infections	228 (13)	432 (20)
Musculoskeletal complaints	149 (9)	130 (6)
Trauma accidental/ intentional	103 (6)	104 (5)
Gastrointestinal diseases	60 (3)	130 (6)
Others^a^	111 (6)	214 (10)
**CHRONIC DISEASES**^b^	8 (< 1)	16 (1)
**TOTAL REFERRALS (accepted by patient)**	38 (2)	46 (2)

^a^acute presentation of medical symptoms, allergies, dental complaints, eye diseases, exhaustion, frostbites, genito-urinary symptoms, wound dressing, malaria

^b^mental health disorders including substances abuse, gastrointestinal system disorder, hepatitis C, diseases of the hematopoietic, urogenital, cardiovascular system, neurological, ophthalmological disorders and chronic lung disease

Among UMC skin conditions (62%, 1059/1718), such as scabies (30%, 520/1718) and body lice (20%, 1055/1718) were the most prevalent. In the group of AMC similarly, most frequent were the skin diseases (51%, 1125/2151), with a slightly lower prevalence of scabies (22%, 476/2151) and body lice (19%, 405/2151). Comparison between UMC and AMC, showed that there was a significant difference in frequencies of main reasons for consultation, with UMC being more affected (*p < 0.001*). The major contribution to the difference in UMC and AMC was related to the following conditions: respiratory infections, skin diseases, other medical conditions, gastrointestinal diseases, MS complaints, trauma and chronic diseases, respectively.

Very few acute traumatic injuries were present in both groups with only 31 (1%) suffering from intentionally caused trauma, while 173 (4.5%) had accidental injuries. Similar low proportions were observed in regard to chronic conditions. Only 2% of the UMC or AMC were referred to internal or external services.

Most UMC patients 32 (84%), were referred to MSF mental health team support due to following causes: symptoms of anxiety, depression, sleeping problems, self-harm, past traumatic experiences including - boat capsizing, attack by police dogs, attempted sexual assault, reported emotional distress, suspected case of torture and victim of torture. The remaining 6 (16%) needed assistance by an external health structure/hospital: emergency medicine department and orthopedic specialist for suspected arm fracture, dentist, hematologist, infectious diseases specialist. Among AMC mental health support was also the most needed as 32 (69.5%) children were recognized for showing psychosomatic symptoms, symptoms of anxiety, depression, insomnia, suspected somnambulism, exhaustion, self-harm, traumatic experiences including beating by police and bombing. 14 (30.5%) were referred to health structure/hospital due to suspected fractures, accidental ingestion of a foreign body, cornea erosion with photophobia, abdominal pain, suspected testicular inflammation, head contusion with hematoma, suspected pregnancy etc.

### Profile and reports of traumatic experiences of patients treated at the mental health department of Belgrade clinic, from January 2018 to January 2019

**Fig. 3:**
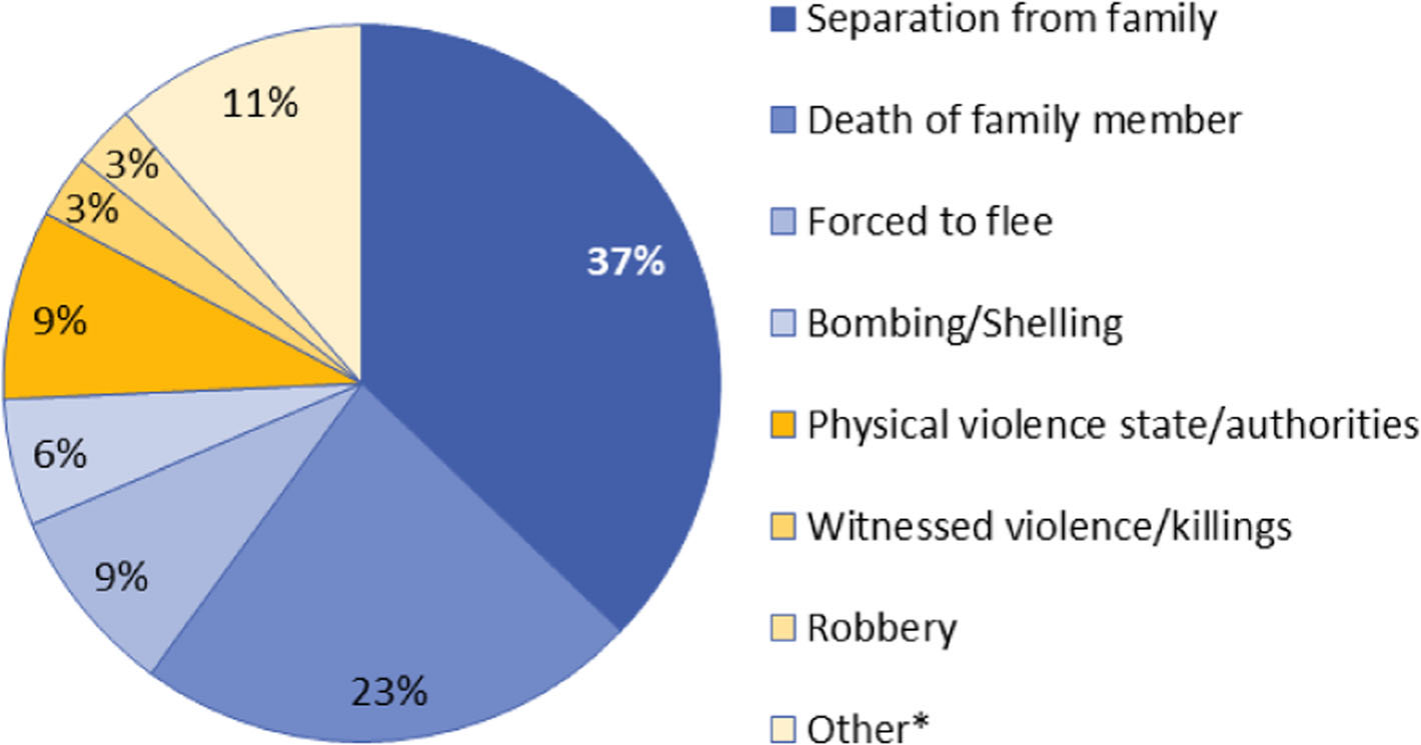
The most frequent traumatic life events reported by unaccompanied migrant children who attended mental health consultations at Belgrade outpatient clinic (*n* = 35). *Other reported traumatic life events included: combat experience, incarceration/kidnapping/hostage, received threats/intimidation. Traumatic events were reported only in those with direct experience. Among UMC 78% reported experiencing a traumatic event and 60% of AMC. For majority of AMC the most traumatic event was experience of physical violence and ill treatment by state authorities (33%, 4/12)

A total of 45 UMC and 21 AMC attended mental health consultations during the study period. Most were from Afghanistan. Almost all UMC (98%) were male in contrast to two thirds of AMC (67%). Both groups were most likely to present with symptoms of anxiety (Table 2). According to clinical evidence, in UMC this symptom was related to separation, sadness and frustration for being away from family and friends (Fig. 3). At the same time anxiety in AMC was largely due to travel-related challenges and uncertainties. As many in this group suffered from behavioral problems, such as acting out, hyperactivity or withdrawal.

**Table 2:** Demographic and mental health characteristics of unaccompanied and accompanied migrant children treated at the Mental health department of Belgrade clinic, from January 2018 to January 2019

MENTAL HEALTH CARE	UMC	AMC
	N (%)	N (%)
**SEX**	45 (100)	21 (100)
Male	44 (98)	14 (67)
Female	1 (2)	7 (33)
**AGE** Median (IQR)	16 (15–17)	15 (11–16.5)
**COUNTRY OF ORIGIN**		
Afghanistan	38 (84)	12 (57)
Pakistan	5 (11)	0
Iran	1 (2)	7 (33)
Bangladesh	1 (2)	0
Palestine	0	1 (5)
North Africa	0	1 (5)
**TRIP DURATION in months** Median (IQR)	21 (16–28)	27 (17–52)
**MAJOR CATEGORY OF MENTAL HEALTH SYMPTOMS**		
Anxiety	10 (22)	5 (24)
Adjustment	8 (18)	2 (10)
Depression	5 (11)	4 (19)
Other^a^	5 (11)	1 (5)
Psychosomatic	5 (1)	0
Post-traumatic stress disorder	2 (4)	2 (10)
Behavioral	1 (2)	5 (24)
Mixed anxiety and depression	1 (2)	0
No mental health symptoms	7 (16)	2 (10)
Victims of torture	4 (9)	3 (14)
Victims of sexual and gender-based violence	2 (4)	2 (9)

^a^sexual problems, self-harm, physical violence survivor, previous diagnosis of mental illness (borderline personality, bipolar disorder) etc.

Those classified as ‘Other’ included issues such as presence of sexual problems, mostly related to lack of knowledge about physiological vs. pathological sexual functioning, having been previously diagnosed with a mental health disorder and self-harm.

17% (11/66) of patients were identified as victims of torture and sexual and gender-based violence, 13% (6/45) among UMC and 24% (5/21) among AMC. The majority of such incidents occurred during migration, with individual cases reporting torture before migration or both pre and during migration period.

Most UMC or 42% (19/45) reported staying in Belgrade streets and squats as compared to AMC 19% (4/21). Among AMC 64% (13/21) resided in government-run asylum and transit reception centers.

Those receiving mental health consultations had been in transit longer (UMC 21 and AMC 27 months) than those who received a medical consultation (in both groups it was 6 months).

### Profile and needs of beneficiaries seeking social care services from January 2018 to January 2019

There were 24 UMC and one AMC that received social services. Among the UMC, 96% were males and 88% were from Afghanistan (Table 3). They had complex and differing case types. Half required medical and social protection and another 38% needed assistance in regard to mental health and social protection. The rest (13%) had requests related to all three types of support (medical, mental health, social protection).

**Table 3:** Demographic characteristics, case type and vulnerabilities in unaccompanied migrant children attending the Belgrade clinic from January 2018 to January 2019

SOCIAL WORKER SERVICES	UMC N (%)
**DEMOGRAPHIC CHARACTERISTICS**	
**SEX**	24 (100)
Male	23 (96)
Female	1 (4)
**COUNTRY OF ORIGIN**	
Afghanistan	21 (88)
Ghana	1 (4)
Iran	1 (4)
Pakistan	1 (4)
**AGE** median (IQR)	16 (14–16)
**ADMINISTRATIVE STATUS**	
Administrative status: Registered	22 (92)
Asylum procedure initiated	4 (17)
**CASE TYPE**	
Social protection w/ Medical care	12 (50)
Social protection w/ Mental health care	9 (38)
Social protection w/ Medical and mental health care	3 (13)
**TYPE OF VULNERABILITIES** (single vs. multiple)	
UMC	7 (29)
UMC and survivor of violence	6 (25)
UMC and survivor of traumatic event, survivor of violence^a^	3 (13)
UMC and survivor of traumatic event	3 (13)
UMC and pregnant woman	1 (4)
UMC and alcohol and drug addiction	1 (4)
UMC and survivor of sexual and gender-based violence, survivor of traumatic event	1 (4)
UMC and survivor of traumatic event, disabled	1 (4)
UMC and survivor of traumatic event, survivor of sexual and gender-based violence, survivor of violence	1 (4)

Note: One AMC who received social assistance has been excluded due to small numbers, ^a^traumatic event refers to witnessing a traumatic event, experiencing separation from family, ill-treatment by authorities, threats/intimidation, death of a family member etc. Violence refers to an incident that includes physical violence

Two thirds of UMC had multiple vulnerabilities (Table 3). Apart from being unaccompanied, 42% were survivors of violence, 38% were survivors of traumatic event(s), 8% were survivors of sexual and gender-based violence (SGBV), one was disabled, one a drug user and one pregnant.

Eighty three percent of UMC required assistance with accessing accommodation and 75% essential needs such as food and non-food items. UMC with history of violence required assistance to access protection and support services (42%). A few (13%) required administrative assistance (Tables 3 and 4). The most frequent provided service was referral to a state centre for social welfare.

**Table 4:** Social needs and outcomes of unaccompanied migrant children attending the Belgrade clinic from January 2018 to January 2019

TYPE OF SOCIAL NEEDS	Services requests N (% of total number of UMC)	Services provided N (% of social care needs)
Total number of UMC	24 (100)	
**ADMINISTRATIVE**		
Administrative assistance	3 (12.5)	3 (100)
**LEGAL**		
Legal assistance	7 (29)	6 (86)
**PROTECTION**		
Referral to state Centre for social work/guardian	23 (96)	23 (100)
Request for accommodation	20 (83)	14 (70)
Safe accommodation^a^	11 (46)	
Accommodation as shelter^b^	10 (42)	
Violence case protection	10 (42)	3 (30)
UNHCR resettlement assistance	4 (17)	0
IOM voluntary return assistance	1 (4)	0
**ESSENTIAL**		
Request for NFI (s)	18 (75)	18 (100)
Request for food	18 (75)	18 (100)
**HEALTH PROTECTION**		
Health services referrals	9 (38)	9 (100)
Accompaniment	9 (38)	8 (89)

^a^ Specialized institution for UMCs and separated children, ^b^ Asylum and Transit-reception centers

## Discussion

### Demographic characteristics, medical and mental health needs

This study described the health profile and social needs of UMC and AMC in the context of transit migration, as seen in Serbia, a country situated along the Western Balkan route. From January 2018 to January 2019 slightly less UMC than AMC visited the Belgrade outpatient clinic. At the clinic in Belgrade, UMC constituted 20% in 2017 and 17% in 2018 of all patients seeking MSF assistance. This was higher than expected as the UN estimated that UMC accounted for 12% of all registered new arrivals in Serbia in 2018 [14]. This indicates that MSF, most probably due to operational and outreach strategy of the project, better managed to reach out to this very vulnerable population. Children with families were generally accommodated in camps and shelters with access to medical services, while MSF operations focused on populations living outside of these facilities, leading to larger proportion of UMC.

The vast majority of migrant children originated from Afghanistan. In comparison to main nationalities observed in government centers, at the MSF clinic, we saw around two times higher proportion of nationals of Afghanistan and a lower proportion of patients from Iran, Iraq, Pakistan and Syria, who are relatively even distributed in the state centers [14–16]. This could imply that some Afghans are more likely to stay outside of the official system, headed by resolve to reach destination sooner and remain more flexible in moving further. High proportion of unaccompanied (51%), Afghan children (81%) was described in a profiling survey of migrants residing in informal sites in Belgrade city center, from 2017. From over 1500 respondents, 78% never went to state accommodation and 62% disclosed they never intended to do so, as their main concern was to leave Serbia and continue toward the EU [17].

By choosing to avoid the official system, many people including children stayed outside, residing in public parks, green areas, car parks, abandoned buildings, areas around railway and bus stations with limited or no access to healthcare and services. Staying in substandard conditions of often overcrowded settlements with changeable access to water and hygienic conditions was one of the main reasons behind high prevalence of skin conditions such as scabies and body lice. Based on our findings, the frequency of these conditions was slightly higher than in similar studies and reports from the EU, which could be attributed to MSF mainly treating individuals who found themselves outside of the official system, in more precarious living environment [18–20].

There was no notable difference in median age between UMC and AMC, however among AMC there was a higher proportion of younger children including those under five, which could explain treating more respiratory and gastro-intestinal conditions, as these are known to be leading causes of morbidity at this age [21].

Among patients who visited a psychologist, anxiety was the most common symptom, while separation anxiety dominated among UMC. Other studies highlighted that having support and advice from adults, during travel, is missed the most by UMC while certain mental health professionals argue that separation from family environment is the main cause of vulnerability of these children [4, 22]. Apart from anxiety, UMC had higher proportion of cases with symptoms of adjustment and PTSD, all related to anxiety disorder symptoms’ spectrum and likely associated with migratory travel struggles. Our results pointing to slightly higher rates of depression and behavioral symptoms among AMC than UMC may be surprising, as this contrasts findings from other research [10]. However, this could be due to several reasons. Influence of family dynamics and mental health status of parents and caregivers is important to child’s mental health along with frequent change of residency or persistent lack of ability to move further, difficulties in establishing and maintaining friendships and relationships other than those with family members [10]. Conversely, UMC usually move in groups and it could be that circumstances during migration help them become more flexible and resilient [22]. Similar research showed that UMC are commonly supported by an adult during journey and some children named peer and ethnic communities, even smugglers as key safeguards while on the move [23].

Whereas UMC and AMC accessed medical care in similar numbers, twice as many UMC received mental health consultations when compared to AMC. In addition, UMC tended to be referred more often, which could infer higher sensitivity of health professionals toward this group, as they are seen to require more support, in contrast to AMC, who are presumed to be in care of their parents or caregivers [24, 25]. Moreover, some children may have been ‘missed’ as MSF on this project does not employ a child psychologist. UMC experienced more traumatic life events, than AMC, which corresponds to accounts of similar studies [10]. Disclosing information about traumatic events is difficult, particularly for children who find themselves in unstable environment, with weak support systems [26]. Our results suggest that experiences of torture and SGBV were slightly higher in AMC group, contrary to preliminary expectations, but likely related to incidents occurring in collective camp settings, where majority of AMC were accommodated.

Trip duration for UMC and AMC attending mental health consultations was over three times longer than those attending medical consultations. This was expected as previous research demonstrated how during migration, uncertainty, lack of control over daily circumstances and long bureaucratic procedures disrupt individual’s mental health [27]. In addition, longer journeys and delays have shown to affect one’s ability to cope with present life situation and often overstretch financial means, adding to their vulnerability [28].

### Social welfare

Needs of migrant children are not only limited to medical and mental health, but include social care needs for accommodation, protection, legal and administrative needs [20]. At the MSF Clinic in Serbia, most requests were related to basic needs such as accommodation, food and NFIs. The high proportion of UMC needing these basic items is unacceptable as ensuring availability of meals, offered with respect to their culture, clothing and shelter are at the core of humanitarian assistance response [22, 29]. Needs assessment studies from destination countries in the EU, emphasize importance of availability of different accommodation options for UMC, based primarily on need, rather than age of the child [26]. Residential specialized institutions for UMC around Serbia have the capacity to accommodate up to 30 children, which represents only a small fraction of those who pass through Serbia [5, 30]. Through the social work program, MSF was effective in bridging the gaps related to accessing the official system and residential accommodation. This was mostly by lobbying and advocating about UMC specific needs, by referring and providing information to beneficiaries.

Difficulties of UMC, in accessing the official system, commonly occur due to their unregulated legal and administrative status [31]. This was important, as our results showed that 92% of UMC were registered with official authorities, hence they have expressed an intention to seek asylum, but few continued with the formal application [32]. In a group of children receiving social support only 17% applied for asylum. Not continuing with the procedure, legally keeps migrants in a ‘gray area’, according to Serbian law. In practice, they could be allowed to stay in accommodation facilities, but their status and rights are not recognized within an official legal framework [33]. For accessing MSF services, administrative status is not an obstacle, as assistance is provided to all, regardless of a person being registered with authorities or not.

As survivors of violence, 42% of UMC requested protection. This higher proportion could be due to MSF strategy to reach out to the most vulnerable, complex cases, or could indicate the vulnerability of UMC to violent incidents [22]. MSF had limited capacities in meeting these needs (only 30%), as procedures are complicated and may require a long period of time to resolve, longer than the length of time a beneficiary may spend in the country. Many survivors of abuse and violence are also reluctant to report incidents mainly due to concerns and trust in the efficacy of the justice system [17].

Several beneficiaries requested assistance regarding resettlement to another country of which none were realized. Serbia is recognized as a safe third country and resettlement process, apart from ‘special protection cases’, has not been running [34]. All beneficiaries went through an official interview process, but during our study period no cases have been resolved.

Generally, MSF provided health services and NFIs directly at the clinic, while legal advice was offered by other collaborating actors. The MSF Clinic in Belgrade city center represents a one stop point for migrants including migrant children who need medical, mental health and social care services. Because they are offered in one place, patients are able to approach and begin solving different physical, psychological and practical problems in one visit. Cooperation between professionals is close and practice of seeing MD as a first contact proved to be positive, as MD usually refers patients further and provides information about available services.

### Recommendations

- Increase capacity and adapt accommodation for UMC. Consider policy and strategy revision to accommodate needs of children in transit, by forming temporary housing capacities close to key areas of congregation in cities, border areas etc.,
- Foster holistic health assessment and services provision. Support distribution of hygiene packs, sleeping bags and clothing to aid treatment of those with skin infestations. Support social workers in developing key skills for working with migrant children and create services that are specific for the children on the move,
- Conduct a more detailed assessment of patients, especially with mental health issues, to estimate closely patient’s existing life situation, needs and accordingly adapt treatment and intervention,
- Establish an improved, joint data collection system at the national and cross-national level, among different stakeholders, to better follow main changes in trends and numbers of UMC and AMC in the country, support their identification, follow up, referral and services provision [22, 23],
- Prioritize needs of AMC in strategic planning and activities, since they can be as significant as those of UMC.

Limitations: Results are representative of the population that visited the MSF clinic only, rather than the whole migrant population present in the country. While cohort of study participants who had a medical consultation was considerable, we did not have a large group of beneficiaries who were referred to social care services. Data used for this research was operational and as such provides limited information.

## Conclusion

Our study shows that unaccompanied and accompanied migrant children have a lot of physical, mental health and social needs. These needs are complex and meeting them in the context of transit migration is difficult. Services need to better adapt by improving access, flexibility, increasing accommodation capacities and training a qualified workforce.

## Data Availability

The data sets are available from the corresponding author on reasonable request.

## Abbreviations

UMC: Unaccompanied Migrant Children
AMC: Accompanied Migrant Children
POM: People on the move
MSF: Médecins Sans Frontières
SGBV: Sexual and gender-based violence
EU: European Union
UN: United Nations
IBM: International Business Machines Corporation
